# Elucidating the Critical Role of Excipients in Gastric Emptying and Oral Absorption of a Rapidly Eliminated BCS I Drug: Implications from Zidovudine Bioequivalence

**DOI:** 10.3390/pharmaceutics18060634

**Published:** 2026-05-22

**Authors:** Yan Lin, Xian Zhang, Fulin Bi, Guangji Wang, Jin Yang

**Affiliations:** 1 School of Basic Medicine and Clinical Pharmacy, China Pharmaceutical University, Nanjing 211198, China; 1020202619@cpu.edu.cn; 2 Center of Drug Metabolism and Pharmacokinetics, China Pharmaceutical University, Nanjing 211198, China; cpuzhangxian@163.com (X.Z.); stu_bfl@163.com (F.B.); 3 Key Laboratory of Drug Metabolism and Pharmacokinetics, China Pharmaceutical University, Nanjing 211198, China

**Keywords:** Biopharmaceutics Classification System Class I (BCS I), biowaiver, excipients, gastric emptying, viscosity

## Abstract

**Background/Objectives:** Despite the presumption of bioequivalence for BCS Class I drugs due to their high solubility and permeability, recent evidence indicates that those with rapid systemic elimination exhibit heightened vulnerability to *C_max_* non-equivalence, primarily attributable to intrasubject variability in gastrointestinal transit and absorption kinetics. It is well known that gastric emptying is a significant physiological-dependent factor. But, does the formulation affect gastric emptying? **Methods:** Using zidovudine as a model drug, formulations containing sodium carboxymethyl starch (CMS-Na), pregelatinized starch, hydroxypropyl methylcellulose (HPMC), and lactose were investigated for their effects on gastric emptying kinetics, and the impact of excipient-mediated gastric emptying prolongation on pharmacokinetic parameters was also evaluated. **Results:** Relative to AZT alone (*C_max_* = 13,350 ng/mL; gastric %ID = 11.3%), co-administration with CMS-Na, pregelatinized starch, or HPMC significantly prolonged gastric retention (%ID: 23.4%, 30.5%, and 40.8% at 22.5 min) and reduced *C_max_* in rats by 47.8%, 34.4%, and 35.1%, respectively, with no effect on intestinal permeability. Viscosity positively correlated with gastric emptying delay. **Conclusions:** Our rat findings provide new possible mechanistic evidence that certain viscosity-modifying excipients can delay gastric emptying and reduce *C_max_* of zidovudine, a rapidly eliminated BCS Class I drug, with potential implications for biowaiver risk assessment. Gastric emptying is not only a physiological-dependent variation but also, in cases where common excipients may significantly delay gastric emptying, a formulation-dependent rate-limiting step. For such drugs, excipient-induced gastric emptying delay poses an underappreciated risk to the biowaiver approach, necessitating more prudent regulatory assessment that encompasses the dynamic interplay among sequential rate processes governing drug disposition.

## 1. Introduction

The oral absorption of a drug is a sequential cascade governed by four kinetic processes: release, gastric emptying, absorption, and systemic clearance. These rates are not independent but exist in a dynamic hierarchy that determines the overall systemic exposure. Crucially, the slowest step in this sequence for a given drug and formulation becomes the rate-limiting step (RLS) that governs the overall pharmacokinetic profile. A change in any upstream rate can propagate downstream, potentially shifting the RLS and altering drug exposure.

**Figure 1:**
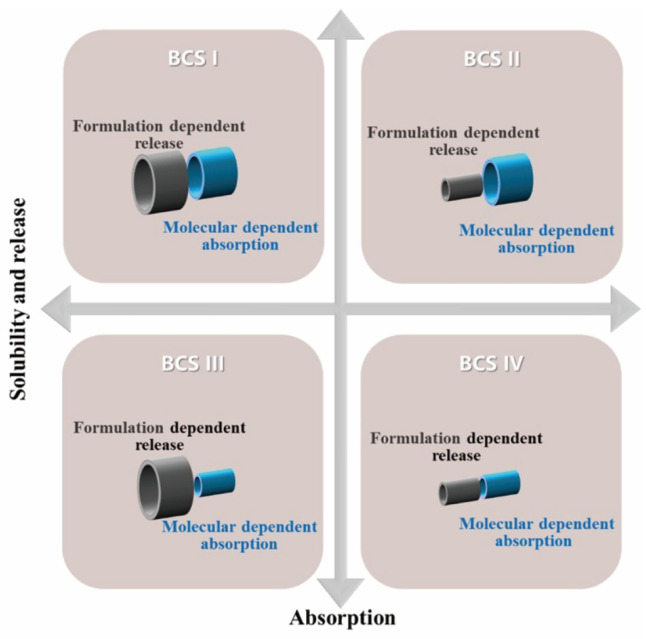
Rate limiting steps for BCS classification.

This framework clarifies the critical distinction between dissolution inequivalence and bioinequivalence. Dissolution inequivalence refers to a measurable difference in the in vitro release rate between two formulations. Bioinequivalence, however, is a clinical outcome defined by a significant difference in the in vivo rate and/or extent of systemic exposure. The conventional Biopharmaceutics Classification System (BCS) paradigm classifies drugs based on which of these two steps (release rate and absorption rate) is inherently rate-limiting (as Figure 1 elegantly depicts as pipes of varying diameters for dissolution and absorption). BCS Class I drugs, with high solubility and permeability, are presumed to have rapid and non-rate-limiting dissolution and absorption [1,2,3]. This justifies biowaivers, as formulation-dependent release differences are not expected to affect systemic exposure. However, recent evidence identifies a critical caveat for a subset of BCS I drugs characterized by rapid systemic clearance [4,5]. Such compounds exhibit heightened vulnerability to peak plasma concentration (*C_max_*) non-equivalence, primarily attributable to intrasubject variability in gastrointestinal (GI) transit and absorption kinetics [4]. Gastric emptying patterns and the cyclical phases of the interdigestive migrating motor complex (MMC) significantly influence the rate and extent of drug presentation to the absorption site; administration during MMC phase I (quiescent period) results in uneven intragastric distribution and delayed absorption, whereas phase II (contractile period) facilitates rapid, homogeneous drug delivery to the duodenum [4]. The slow gastric emptying rate and the rapid drug release are the root causes for the exemption of BSC1 class drugs. For BCS Class I drugs, gastric emptying is generally regarded as a physiological covariate whose variability can be effectively controlled for in a standard crossover bioequivalence (BE) study design, thereby supporting the rationale for a biowaiver. When systemic clearance is exceptionally fast, gastric emptying—often assumed to be a rapid and purely physiological covariate—can emerge as a de facto rate-controlling step. Even minor perturbations in gastric emptying kinetics can significantly influence the rate of drug absorption, potentially leading to bioinequivalence.

The research on the generic drugs of dexketoprofen (a rapidly eliminated BCS Class I drug) provides a typical example. The first two batches of the dexketoprofen preparations had equivalent dissolution rates but showed bioinequivalence, while the third batch showed the opposite pattern—the dissolution rates were not equivalent but showed bioequivalence [6]. This suggests that the observed bioinequivalence was attributable to intrinsic formulation factors (differences in formulation composition or manufacturing processes). To explore the underlying mechanisms, we previously conducted a PBPK modeling and simulation study [5], which demonstrated that for dexketoprofen trometamol, a rapidly eliminated BCS Class I drug, liquid gastric emptying time is a sensitive parameter affecting *C_max_* under biowaiver conditions. Their virtual BE studies revealed that while dissolution rate changes within biowaiver boundaries did not cause high non-BE ratios, excipient effects on gastric emptying remain an uncharacterized risk factor requiring experimental investigation. The present study addresses this gap by providing direct mechanistic evidence in rats, aiming to investigate the influence of liquid gastric emptying and permeability on the pharmacokinetics of a rapidly eliminated BCS Class I drug.

Due to species differences in the metabolism of dexketoprofen, zidovudine (AZT), a BCS Class I compound with rapid elimination in both humans and rats [7,8,9], was selected as the model drug for further investigation owing to its narrow therapeutic window and reported *C_max_* variability in BE studies [10,11]. Based on the excipient composition comparison between marketed generic and reference-listed zidovudine products, four commonly used excipients—sodium carboxymethyl starch (CMS-Na), pregelatinized starch, hydroxypropyl methylcellulose (HPMC), and lactose, serving distinct functional roles as superdisintegrants, diluents/binders, controlled-release matrices, and fillers, respectively—were selected to investigate their individual effects on gastric emptying and absorption of zidovudine, as well as their overall influence on the pharmacokinetic (PK) profile in rats [12]. Understanding their potential impact on the absorption of rapidly eliminated BCS I drugs is critical for rational formulation design and BE assessment.

Therefore, the specific objectives for this study were

(1) To quantitatively assess the effects of these excipients on liquid gastric emptying kinetics using dynamic ^18^F-FDG PET/CT imaging in rats and correlate findings with excipient viscosity;
(2) To evaluate the impact of each excipient on the systemic exposure of zidovudine through pharmacokinetic studies in rats;
(3) To elucidate the primary mechanism by determining whether these excipients directly influence the intestinal permeability of zidovudine using Caco-2 transport assays.

Our study elevates the theoretical understanding of oral drug absorption by introducing gastric emptying as a critical, excipient-sensitive node in the absorption cascade for BCS I rapidly eliminated drugs. It provides a mechanistic rationale for BE failures in this subclass and argues for a more nuanced, physiology-informed application of biowaiver principles, suggesting that the “high solubility—high permeability” criteria, while necessary, may not be sufficient to guarantee formulation interchangeability for all BCS Class I drugs.

## 2. Materials and Methods

### 2.1. Materials

Zidovudine (300 mg tablets, batch no. C1826133) was obtained from Shanghai Aladdin Biochemical Technology Co., Ltd., Shanghai, China. The excipients—sodium carboxymethyl starch (CMS-Na, batch no. G31A11F122825), pregelatinized starch (batch no. Z10O11W126822), hydroxypropyl methylcellulose (HPMC, batch no. C12563774), and lactose (batch no. Q23F12N139784)—were supplied by Shanghai Yuanye Bio-Technology Co., Ltd., Shanghai, China with the exception of HPMC, which was sourced from Shanghai Macklin Biochemical Co., Ltd., Shanghai, China, ^18^F-FDG was procured from Nanjing Jiangyuan Andike Positron Research and Development Co., Ltd., Nanjing, China. The Caco-2 cell line was acquired from the Chinese Academy of Sciences (Shanghai, China).

### 2.2. LC-MS/MS Bioanalytical Method of AZT

A reliable and sensitive LC-MS/MS method was developed and validated for the quantification of AZT in rat plasma and Hanks’ balanced salt solution (HBSS) matrix to support subsequent studies on the effects of excipients on its oral absorption in rats and transport across Caco-2 cell monolayers. Sample preparation was performed using protein precipitation for both biological and buffer matrices. Chromatographic separation was achieved on a Phenomenex Synergi Hydro-RP 80Å column (50 mm × 2.0 mm, 4 μm) maintained at 35 °C, with an injection volume of 3 μL and a flow rate of 0.6 mL/min. The mobile phase consisted of (A) 0.3% formic acid in water and (B) 0.3% formic acid in methanol, using a gradient elution. The gradient elution program was as follows: 0–0.1 min, 20% B; 0.1–2.0 min, linear increase to 55% B; 2.0–2.1 min, linear increase to 95% B; 2.1–3.2 min, hold at 95% B; 3.2–3.3 min, decrease to 20% B; and 3.3–4.5 min, re-equilibration at 20% B. The total run time was 4.5 min per injection, and the injection volume was 3 μL.

Mass spectrometric detection was performed on an API 4000 triple quadrupole mass spectrometer (AB Sciex, Framingham, MA, USA) equipped with an electrospray ionization (ESI) source operating in positive ion mode. Multiple reaction monitoring (MRM) transitions were *m*/*z* 268.2 → 127.0 for zidovudine and *m*/*z* 340.3 → 116.1 for alogliptin (internal standard, IS). The optimized source parameters were ion spray voltage, 4500 V; source temperature, 450 °C; curtain gas (CUR), 10 psi; collision gas (CAD), 6 psi; nebulizer gas (GS1), 45 psi; and heater gas (GS2), 40 psi. Compound-dependent parameters were declustering potential (DP), 48 V (AZT) and 75 V (IS); collision energy (CE), 14 eV (AZT) and 43 eV (IS); collision cell exit potential (CXP), 7 V (AZT) and 6 V (IS); and entrance potential (EP), 10 V for both analytes. Dwell time was 300 ms per transition.

Sample preparation employed protein precipitation. Briefly, 20 μL of plasma or HBSS sample was mixed with 20 μL of IS working solution (1 μg/mL alogliptin in methanol) and vortexed for 1 min. Then, 320 μL of methanol was added as the precipitation solvent, and the mixture was vortexed for 8 min, followed by centrifugation at 12,000 rpm for 6 min at 4 °C. An 80 μL aliquot of the supernatant was diluted with 320 μL of water, vortexed for 8 min, and 200 μL was injected into the LC-MS/MS system. This 1:16 precipitation ratio was optimized to achieve maximal protein removal while maintaining sufficient analyte sensitivity.

### 2.3. PET/CT Imaging Study for Gastric Emptying Evaluation

Thirty male Sprague Dawley rats were purchased from the same vendor batch and acclimatized for 7 days under identical housing conditions. Prior to the experiment, animals were randomized to treatment groups using a computer-generated random number sequence (Excel RAND function), stratified by body weight to ensure comparable mean weights across groups. The randomization sequence was generated by an independent investigator not involved in animal handling or data collection.

A parallel study design was employed in which rats fasted overnight received a single oral gavage administration of zidovudine in one of five formulations (*n* = 6 per group): AZT alone (control), AZT with 5% CMS-Na, AZT with 3% pregelatinized starch, AZT with 4% HPMC, or AZT with 3% lactose (see Table 1 for details about the groups and dosing). Each formulation included the tracer ^18^F-FDG at a target radioactivity of approximately 600 μCi per rat, with the actual dose adjusted based on individual body weight and specific activity.

Dynamic small-animal PET imaging of the thoracoabdominal region (covering the stomach and intestines) was initiated immediately after ^18^F-FDG administration and continued for 60 min. The energy window for acquisition was set to 350–650 keV. The acquired images were reconstructed, and subsequent analysis was conducted using PMOD software version 3.8 to determine the radioactivity concentration (radioactivity per unit volume) within regions of interest (ROIs). ROIs were manually delineated on co-registered PET/CT images using PMOD software. The stomach ROI was anatomically defined on CT images (fundus, body, and antrum) and transferred to PET. The use of co-registered CT anatomical guidance ensured robust separation of the stomach and intestine. The operator performing image reconstruction and ROI analysis was blinded to the group allocation; data files were coded with animal ID numbers that did not reveal treatment assignment. ROIs were drawn by the experienced operator trained according to the internal SOP (Standard of Practice).

The percentage of injected dose per gram of tissue (%ID/g) was calculated according to the following procedure. First, the administered radioactivity was decay-corrected from the initial dose calibration time (*t*_0_) to the image acquisition time (*t*) using the physical half-life of ^18^F (110 min).

$$A_{Corrected}=A_{0}\times 0.5^{\frac{t-t_{0}}{T_{1/2}}}$$

where *A*_0_ is the initial calibrated activity and *T*_1_/_2_ is the physical half-life of ^18^F. This correction accounts for radioactive decay between dose preparation and imaging.

Second, the %ID/g was computed by dividing the decay-corrected radioactivity concentration in the region of interest (μCi/g) by the total injected dose (μCi) and multiplying by 100%. This normalization approach is standard in preclinical PET imaging and allows for direct comparison of gastric retention across animals receiving slightly different injected doses.

$$\%ID/g=\frac{Radioactivity\;Concentration\;in\;the\;Region\;of\;Interest\;(\mu Ci/g)}{Injected\;Dose\;(\mu Ci)}\;\times 100\%$$

Gastric emptying data were fitted to a first-order kinetic model. Additionally, the viscosity of each formulation was measured using a DV2T viscometer (Brookfield, AMETEK Inc., Chandler, AZ, USA).

### 2.4. Pharmacokinetic Study in Rats

The effects of the four excipients on the oral absorption of zidovudine were investigated in rats. This study employed five experimental groups (*n* = 6 per group), consistent with the grouping scheme used in the PET/CT gastric emptying study. Each rat received zidovudine at a dose of 27 mg/kg. Following a 12 h fast, the formulations were administered by oral gavage. Blood samples were collected from the orbital plexus pre-dose and at 10, 15, 20, 25, 30, and 35 min, as well as 1, 2, 4, and 8 h post-dose. Plasma concentrations of zidovudine were determined immediately after sample collection using the developed LC-MS/MS method.

A noncompartmental analysis was performed using Phoenix WinNonlin^®^ 8.2 to estimate the following pharmacokinetic parameters: *C_max_*, area under the plasma concentration–time curve from 0 to 8 h (*AUC*_0–8h_), time to reach *C_max_* (*T_max_*), and elimination half-life (*T*_1/2_). *C_max_* and *T_max_* were determined directly from the observed concentration–time data without interpolation. The terminal elimination phase was defined by visual inspection of the log-linear terminal portion of the plasma concentration–time curve, requiring a minimum of three data points for the slope estimation. For AZT, the terminal elimination half-life in control rats was approximately 0.62 h; given that 8 h corresponds to approximately 13 half-lives, this sampling duration ensures that the extrapolated AUC fraction (*AUC*_0–∞_ − *AUC*_0–8_)/*AUC*_0–∞_ was <5% for all groups, thereby minimizing the impact of truncation error on AUC estimation. Late-time concentrations at 8 h remained above the lower limit of quantification (30 ng/mL) in most groups. Statistical comparisons of key pharmacokinetic parameters between groups were conducted using one-way ANOVA in GraphPad Prism 6, with a significance level set at *p* < 0.05.

### 2.5. Caco-2 Cell Permeability Study

Caco-2 cells were seeded on polyester Transwell^®^ inserts (0.3 cm^2^ growth area and 1.0 μm pore size) at a density of 2 × 10^5^ cells/mL and cultured for 21 days with complete medium changes every 2 days for the first week and daily thereafter. Monolayer integrity was monitored by transepithelial electrical resistance (TEER) measured with a Millicell^®^ ERS-2 electrode. Transport experiments were conducted in HBSS (pH 7.4, supplemented with 25 mM glucose and 10 mM HEPES) at 37 °C with orbital shaking at 50 rpm. The apical (AP) pH was 7.4, and the basolateral (BL) pH was 7.4.

The experiment included five treatment groups, consistent with the in vivo study design. Donor solutions contained AZT at 50 μg/mL (186 μM), alone or with excipients at the following concentrations: CMS-Na (20 μg/mL), HPMC (20 μg/mL), pregelatinized starch (20 μg/mL), or lactose (30 μg/mL). These concentrations were selected based on the survival rate of Caco-2 cells in different concentration administration groups. In addition, the Caco-2 concentrations employed in this study (20–30 μg/mL) were deliberately selected to be substantially below these estimated luminal levels. This conservative approach is consistent with established guidance for excipient permeability screening [13,14], which posits that if no permeability-modulating effect is observed at concentrations below anticipated luminal levels, it is unlikely that such an effect would manifest in vivo at higher concentrations. This strengthens the negative predictive value of our Caco-2 findings. This approach ensures that any lack of observed effect in vitro is not attributable to concentration-dependent saturation of excipient–membrane interactions.

For apical-to-basolateral (A-B) transport, 200 µL of the test solution was applied to the apical chamber, while for basolateral-to-apical (B-A) transport, 1 mL was added to the basolateral chamber. Samples (100 µL) were collected from the receiver compartment at 0.5, 1, 1.5, and 2 h.

The apparent permeability coefficient (Papp, cm/s) was calculated as [15]

Papp = (*dQ*/*dt*)/(*A* × *C*_0_)

where *dQ*/*dt* is the transport rate (ng/s or nM/s), *A* is the membrane surface area (cm^2^), and *C*_0_ is the initial concentration (ng/mL or nM/mL).

The efflux ratio (ER) was determined as [15]

ER = (Papp (B − A)/Papp (A − B)) ×100%

An ER value greater than 2 suggests potential involvement of efflux transporters.

Data were analyzed using one-way analysis of variance (ANOVA).

## 3. Results

### 3.1. PET/CT Imaging Reveals Excipient-Induced Delayed Gastric Emptying

PET/CT imaging after oral coadministration of ^18^F-FDG with zidovudine alone or in combination with one of the four excipients (CMS-Na, pregelatinized starch, HPMC, or lactose) revealed significant differences in the gastric emptying rates. At 1350 s (22.5 min) post-dosing, the mean gastric radioactive %ID values were 11.3% (G1: AZT control), 23.4% (G2: AZT + CMS-Na), 30.5% (G3: AZT + pregelatinized starch), 40.8% (G4: AZT + HPMC), and 17.1% (G5: AZT + lactose). These values correspond to a gastric emptying rate order of G1 > G5 > G2 > G3 > G4. Consistent with this pattern, intestinal %ID values in the control group (G1) were consistently higher than those in all excipient groups, indicating more rapid transit of the liquid formulation from the stomach to the intestine in the absence of viscosity-modifying polymers.

**Figure 2:**
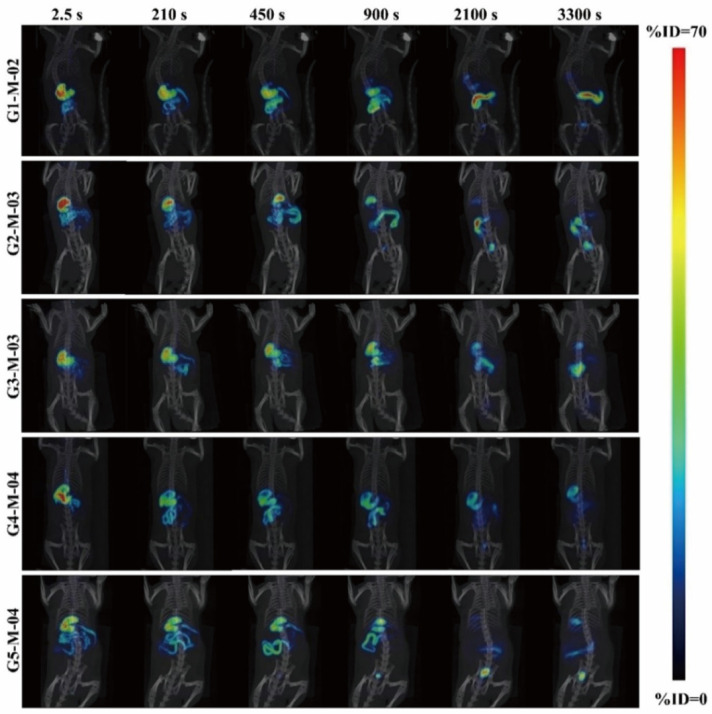
FiMIP images of PET/CT scans of representative animals in each group after a single intragastric administration of zidovudine and four excipients.

**Figure 3:**
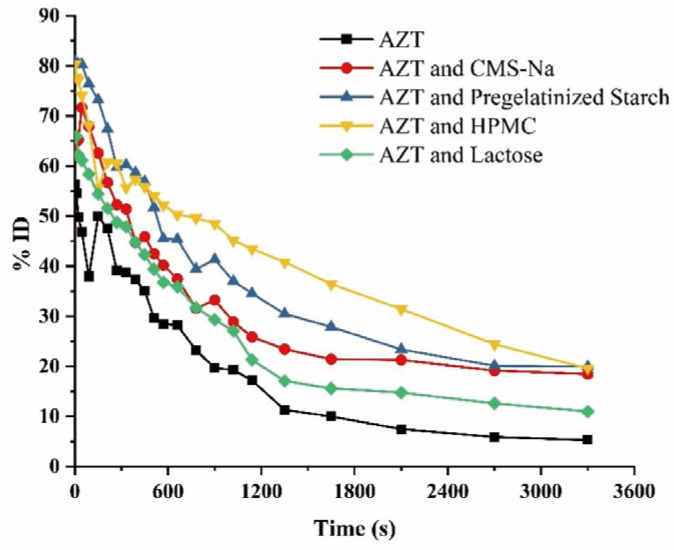
The radioactive %ID value (mean) of the stomach of each group after a single gavage of zidovudine and four excipients in rats.

**Figure 4:**
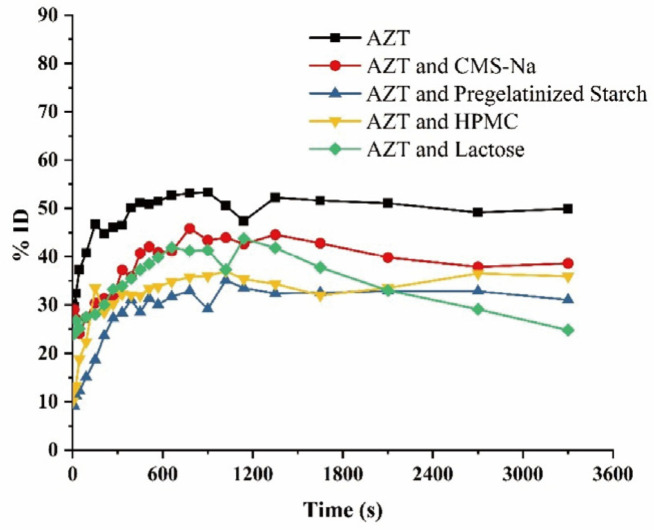
The radioactive %ID value (mean) of the intestine of each group after a single gavage of zidovudine and four excipients in rats.

The mean radioactive %ID values in the stomach and intestine at different time points are summarized in Table 2 and Table 3, respectively. Based on two-way ANOVA, %ID values in the stomach in rats among different groups did have a statistical difference (*p* < 0.0001). Dunnett’s multiple comparison test also showed that different excipients indeed affect gastric emptying in rats compared with the G1 group (see the Supplementary Materials, Table S1). Representative maximum intensity projection (MIP) images from PET/CT scans are shown in Figure 2, and the time profiles of gastric and intestinal %ID are presented in Figure 3 and Figure 4.

**Figure 5:**
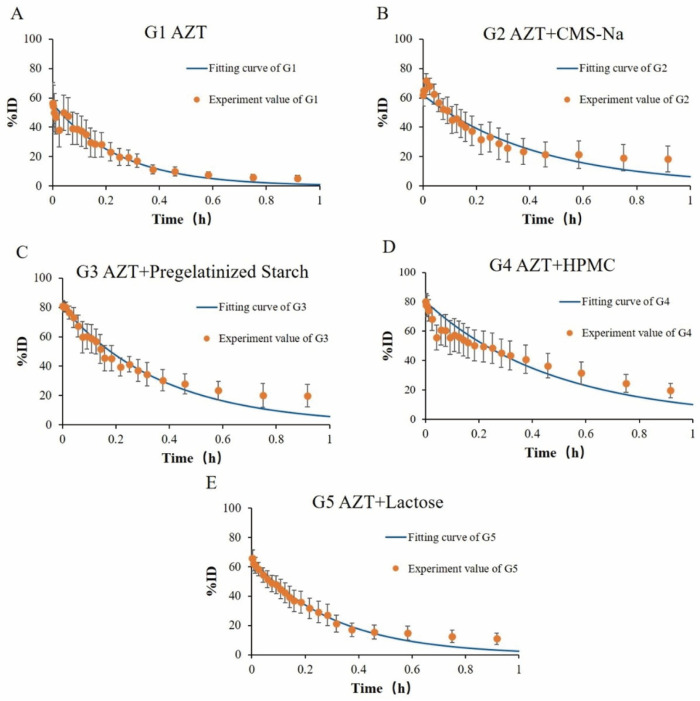
Kinetic fitting curve using mean %ID experimental value for (**A**) taking AZT, (**B**) taking AZT and CMS-Na, (**C**) taking AZT and pregelatinized starch, (**D**) taking AZT and HPMC, and (**E**) taking AZT and lactose.

The average values of gastric emptying data in each group were fitted to a first-order kinetic model (Figure 5 and Table 4), revealing the following gastric emptying rate constants: control (4.01 h^−1^) > lactose (3.3 h^−1^) > CMS-Na (2.3 h^−1^) ≈ pregelatinized starch (2.67 h^−1^) > HPMC (2.09 h^−1^). While the rate constant for the lactose group (G5) was only slightly lower than that of the control (G1), significant reductions were observed in groups containing CMS-Na (G2), pregelatinized starch (G3), and HPMC (G4). These results suggest that CMS-Na, pre-gelatinized starch, and HPMC may delay fluid gastric emptying in rats. Individual PET gastric emptying exponential fitting and distribution, and summary statistics of rate constant *k* can be found in the Supplementary Materials, Figures S2 and S3.

Viscosity was measured using a DV2T viscometer with different rotational speeds (RPM) across groups because the formulations had markedly different viscosities and the RPM for each group was selected to ensure that the torque remained within the instrument’s optimal range (10–100%). Operating outside this range compromises measurement accuracy. Consequently, low-viscosity solutions (AZT control, lactose, and pregelatinized starch) were measured at 200 RPM, the intermediate-viscosity CMS-Na solution at 30 RPM, and the high-viscosity HPMC solution at 10 RPM. Under these conditions, each measured viscosity value is precise and reliable. However, since CMS-Na, pregelatinized starch, and HPMC are macromolecular excipients whose aqueous systems exhibit concentration-dependent non-Newtonian behavior, their apparent viscosities are inherently shear-rate-dependent. This is a limitation of the present study. Future work should measure viscosity at identical shear rates (e.g., same RPM and spindle) or, preferably, construct full flow curves (viscosity vs. shear rate) to enable meaningful comparison and to better predict the flow behavior under physiological or processing conditions.

Viscosity measurements revealed the following rank order among the formulations: AZT + HPMC > AZT + CMS-Na > AZT + pregelatinized starch > AZT + lactose ≈ AZT control (as in Table 5). The rank order of viscosity was generally aligned with the rank order of gastric emptying delay observed in PET imaging (AZT + HPMC > AZT + CMS-Na > AZT + pregelatinized starch > AZT + lactose > control) and *C_max_* reduction (AZT + CMS-Na > AZT + HPMC ≈ AZT + pregelatinized starch > AZT + lactose). The direct correlation between measured viscosity and the extent of gastric emptying delay suggests that increased liquid viscosity is the primary mechanism responsible for the observed retardation in gastric emptying. To quantitatively assess the relationship between formulation viscosity and gastric emptying delay, a Spearman correlation analysis was performed between the apparent viscosity and the fitted first-order gastric emptying rate constants (*k*). A strong negative correlation was observed (*r* = −1.000, *p* = 0.0167, *n* = 5), indicating that higher-viscosity formulations were associated with slower gastric emptying (see the Supplementary Materials, Table S2).

### 3.2. In Vivo Pharmacokinetic Study: C_max_ Reduction Consistent with Delayed Gastric Emptying

**Figure 6:**
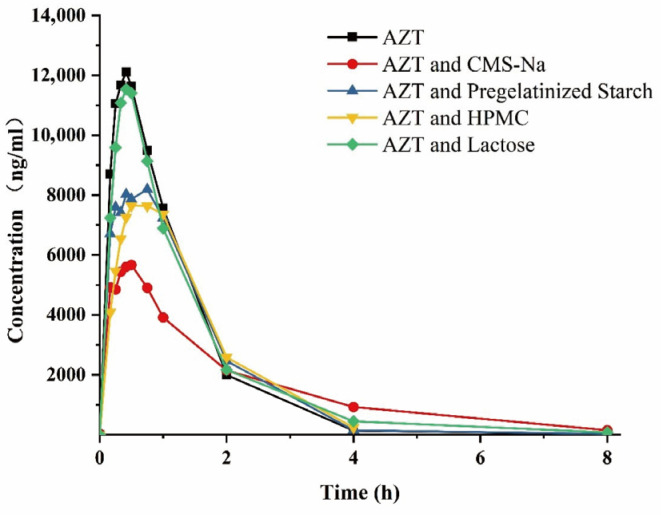
Mean plasma AZT concentration–time profiles following oral administration of AZT alone or with excipients.

Plasma concentration–time profiles of zidovudine were determined in rats following the administration of AZT alone or with excipients (Figure 6). The results revealed marked alterations in the pharmacokinetic curves when AZT was co-administered with viscosity-enhancing excipients. While the lactose group showed nearly identical absorption characteristics to the control, the three polymer-containing groups exhibited substantially reduced peak plasma concentrations (*C_max_*). The most pronounced decrease in *C_max_* was observed in the CMS-Na group, followed by the HPMC and pregelatinized starch groups.

The pharmacokinetic parameters derived from noncompartmental analysis are summarized in Table 6. One-way ANOVA results were reported on the last column. The *C_max_* values show statistical differences among groups (*p* = 0.04). The mean *C_max_* values for the AZT + CMS-Na, AZT + pregelatinized starch, and AZT + HPMC groups were 6965.00, 8760.00, and 8658.33 ng/mL, respectively, all substantially lower than that of the AZT control group (13,350.00 ng/mL). The rank order of *C_max_* reduction observed in the rat pharmacokinetic study was AZT + CMS-Na > AZT + HPMC ≈ AZT + pregelatinized starch > AZT + lactose. This sequence correlated closely with the viscosity ranking of the formulations, although the *C_max_* suppression in the AZT + CMS-Na group appeared more pronounced than anticipated based solely on viscosity measurements. Statistical analysis confirmed a significant reduction in *C_max_* for the AZT + CMS-Na group compared to the control based on a post hoc analysis (*p* < 0.05). In contrast, the AZT + lactose group showed a *C_max_* value (12,305.00 ng/mL) comparable to the control. The *AUC*_0–8_ and *AUC*_0–∞_ values were not statistically different between any excipient group and the control (all *p* > 0.05), supporting the conclusion that the extent of AZT absorption was unaffected by the excipients. These data confirm that viscosity-modifying excipients alter the rate but not the extent of absorption for zidovudine.

Although the rank order of gastric emptying delay generally followed formulation viscosity, CMS-Na induced a greater reduction in *C_max_* (47.8% relative to the control) than would be anticipated from its intermediate viscosity position (38.4 cP). This deviation suggests that factors beyond bulk viscosity contribute to the CMS-Na effect. First, CMS-Na is an anionic polyelectrolyte that undergoes rapid swelling upon contact with aqueous fluids [16]. At the concentration used (5%, *w*/*v*), partial gelation or the formation of a viscoelastic layer at the gastric interface may have occurred, creating a physical barrier to transpyloric flow that is not captured by a single-point viscosity measurement at 30 RPM. Second, negatively charged carboxymethyl groups can electrostatically interact with the positively charged gastric mucus layer, conferring mucoadhesive properties to CMS-Na [17,18]. Such interactions could prolong gastric residence beyond the effect of bulk viscosity alone. Third, CMS-Na exhibits pronounced shear-thinning behavior; the viscosity measured at 30 RPM (moderate shear) likely underestimates its resistance to flow under the low-shear conditions prevailing in the resting stomach, where the effective viscosity may be substantially higher [19]. Together, these physicochemical properties—gelation tendency, mucoadhesion, and shear-dependent rheology—modulate the magnitude of gastric emptying delay and can explain why CMS-Na produced a *C_max_* reduction approaching that of the more viscous HPMC formulation despite its intermediate single-point viscosity.

Elimination parameters revealed a significantly prolonged half-life (*T*_1/2_) in the AZT + CMS-Na group compared to the control, while other excipient groups showed no notable differences in elimination characteristics. This phenomenon could theoretically reflect either (i) a true change in elimination clearance or (ii) flip-flop kinetics, where the prolonged absorption input (due to gastric emptying delay) becomes rate-limiting and determines the apparent terminal slope [20]. Given that (a) CMS-Na is not known to inhibit AZT metabolism, (b) the unchanged AUC and delayed *T_max_* in the CMS-Na group support flip-flop kinetics rather than altered systemic elimination, and (c) the PET/CT data confirmed marked gastric retention in the CMS-Na group, we attribute the apparent *T*_1/2_ prolongation primarily to flip-flop kinetics secondary to delayed gastric emptying rather than to altered elimination.

### 3.3. Caco-2 Study: Excipients Do Not Affect Permeability

Monolayer integrity was monitored by TEER measured with a Millicell^®^ ERS-2 electrode. Only monolayers with TEER values ≥ 500 Ω·cm^2^ on day 21 were used for transport experiments; the mean TEER across all experiments was 520.1 ± 29.2 Ω·cm^2^ (see the Supplementary Materials, Table S3).

Paracellular permeability was assessed using fluorescein (Lucifer yellow, 300 μM), a marker of tight junction integrity. The apparent permeability coefficient (Papp) of fluorescein was 2.73 ± 0.87 × 10^−8^ cm/s, well below the acceptance threshold of 1 × 10^−7^ cm/s, confirming intact tight junctions. Positive control permeability markers were tested in each experimental batch to ensure system suitability. The rank order (propranolol > metoprolol > atenolol) and absolute values were consistent with literature data and BCS permeability classifications (see the Supplementary Materials, Table S4).

**Figure 7:**
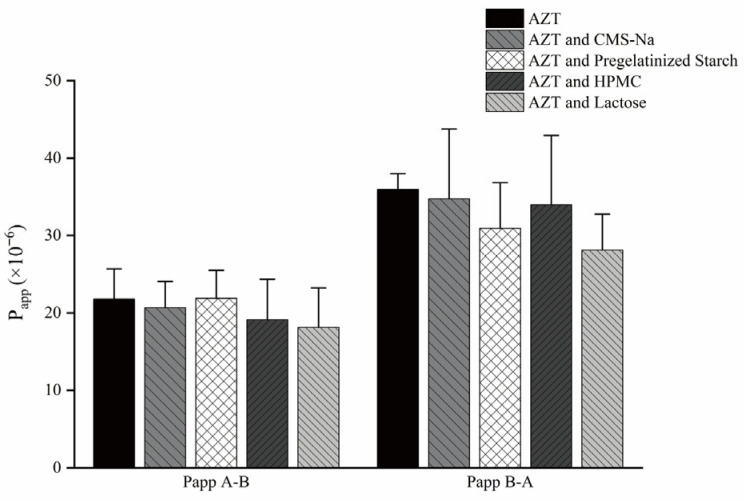
The Papp values of AZT and four excipients in the Caco-2 cell model.

In the Caco-2 cell model, zidovudine alone exhibited a Papp (A-B) value of (21.80 ± 3.88) × 10^−6^ cm/s and a Papp (B-A) value of (35.94 ± 2.05) × 10^−6^ cm/s, yielding an efflux ratio (ER) of 1.65, which indicates that zidovudine is not a substrate for efflux transporters. As summarized in Table 7 and Figure 7, co-administration with each of the four excipients did not significantly alter the apparent permeability coefficients in either direction. One-way ANOVA confirmed no statistically significant differences in either Papp (A-B) or Papp (B-A) among the groups, demonstrating that the membrane permeability of zidovudine remains unaffected by the presence of these excipients (for details of the post hoc analysis among groups, see the Supplementary Materials, Table S5).

## 4. Discussion

It is widely accepted that conventional pharmaceutical excipients exert negligible effects on gastric emptying rate, particularly for BCS Class I compounds characterized by high solubility and high permeability [21,22]. However, this prevailing view may underestimate the potential of excipients to induce bioinequivalence under specific circumstances [22,23]. Although the stomach is not a primary site of drug absorption for the vast majority of pharmaceuticals, gastric emptying governs the onset of drug entry into the intestine, where absorption commences. Excipients are generally present in much larger quantities than the active pharmaceutical ingredient (API), and their amounts can vary substantially across different formulations. While traditionally regarded as pharmacologically inert, a growing body of evidence indicates that excipients can substantially modulate drug absorption through distinct mechanisms: (1) inhibition of efflux transporters such as P-glycoprotein (P-gp) and breast cancer resistance protein (BCRP) [24,25], and (2) osmotic acceleration of GI transit [26]. Nevertheless, current research has largely overlooked their potential impact on gastric emptying time. More critically, although previous modeling studies have identified gastric emptying as a key risk factor for biowaiver approval of rapidly eliminated BCS Class I drugs [5], direct experimental evidence is lacking to quantitatively assess how specific pharmaceutical excipients modulate in vivo gastric emptying kinetics.

Common methods for assessing gastric emptying include scintigraphy [27], ^13^C-labeled breath tests [28], ultrasonography [29], wireless motility capsules, and positron emission tomography (PET) [30]. As a highly sensitive, non-invasive technology, PET is particularly suitable for in vivo drug analysis, especially for quantifying drug concentrations in tissues. Previous studies have demonstrated the feasibility of using PET to measure gastric emptying and evaluate gastrointestinal absorption processes [30,31]. This study employed dynamic and visual PET/CT imaging to directly and quantitatively evaluate the impact of commonly used excipients on gastric emptying kinetics and to investigate their causal relationship with the in vivo absorption behavior of BCS Class I drugs. The objective was to provide mechanistic insights and experimental evidence essential for refining the biowaiver strategy for high-risk BCS Class I compounds.

^18^F-FDG was selected as the PET tracer—a well-established and clinically widely used probe with a confirmed safety profile [32,33,34]. ^18^F-FDG is a glucose analog that is transported into cells via GLUT transporters and subsequently phosphorylated by hexokinase, raising concerns that tissue uptake may confound luminal quantification [30]. However, during the acute phase (0–60 min) following oral administration, the signal predominantly reflects intraluminal content because gastric emptying is the rate-limiting step for luminal transit [30,35]. Identical ^18^F-FDG dosing and scanning protocols across groups ensure any tissue uptake is systematically distributed, preserving the validity of relative between-group comparisons. We acknowledge that physiological FDG uptake in gastric musculature and intestinal mucosa (mediated by GLUT2/GLUT5) becomes increasingly relevant at >60 min; accordingly, kinetic modeling was restricted to 0–60 min, and animals were fasted overnight to minimize basal metabolic activity. While non-absorbable tracers (e.g., ^68^Ga-NOTA) offer theoretical advantages [31], ^18^F-FDG has been validated for gastrointestinal transit assessment and was employed here under controlled conditions that mitigate tissue uptake bias.

In the present PET study, the 22.5 min time point was selected as a representative reference time for comparing the effects of pharmaceutical excipients on gastric emptying in rats under the experimental conditions employed. Published data on gastric half-emptying time (*T*_½_g_) in rats indicate considerable variability depending on meal type, consistency, and physiological state, with reported *T*_½_g_ values typically ranging from approximately 7 to 138 min [36]. For solid or semi-solid meals in fasted or normal rats, half-emptying values frequently fall between 15 and 30 min; for instance, Kirman et al. (2012) reported a *T*_½_g_ value of 17 min in rats [36]. In a recent PET imaging study in small animals, Chen et al. (2021) reported that the half-emptying times for four different test meals in mice ranged from 3.92 ± 0.87 min to 59.7 ± 3.11 min [31]. Taken together, 22.5 min approximates the midpoint in the typical *T*_½_g_ range covering most liquid and semi-solid meals and thus served as a harmonized reference time for quantifying gastric retention under the present PET protocol. It should be noted, however, that *T*_½_g_ is a population-based summary statistic; the ranking of gastric emptying rates among different treatment groups may vary at specific early or late time points. Therefore, the 22.5 min time point is used for standardized inter-group comparison rather than implying a universal emptying order across all groups at all measurement points.

Dynamic PET data were acquired for a total duration of 3300 s (55 min). This acquisition window was designed to fully capture the gastric emptying process based on previously established small-animal PET methodologies. Yamashita et al. (2011) employed a similar dynamic PET protocol to assess gastrointestinal absorption of ^18^F-FDG in rats and demonstrated that the gastric emptying phase could be adequately characterized within 60 min of dynamic scanning [30]. Acquisition extended beyond 60 min is constrained by the effective half-life of the PET radiotracer, which accounts for both physical decay and biological clearance. Scanning beyond 60 min would result in substantially decreased counting statistics, compromising the reliability of time-activity curve fitting. Accordingly, 3300 s was selected as the upper bound for dynamic acquisition in accordance with standard small-animal PET gastric emptying protocols, ensuring sufficient signal-to-noise ratio for accurate kinetic analysis while avoiding unnecessary extension beyond the useful timeframe of the tracer.

This study systematically evaluated the impact of four excipients—sodium carboxymethyl starch (CMS-Na), pregelatinized starch, HPMC, and lactose—on gastric emptying and the pharmacokinetics of zidovudine in rats. These excipients were selected based on two criteria: exclusion of those identical to the reference formulation and exclusion of insoluble materials, given that liquid gastric emptying rate was the sensitivity parameter of interest. The excipient concentrations were selected based on a three-tier rationale: (i) typical use levels in marketed immediate-release tablet formulations as documented in the Handbook of Pharmaceutical Excipients and regulatory pharmacopeias; (ii) the solubility limits of each excipient in physiological saline, ensuring administrability by oral gavage; and (iii) the need to achieve a physiologically relevant range of viscosities to test the viscosity–gastric emptying hypothesis mechanistically.

The results demonstrated varying degrees of gastric emptying delay induced by the different excipients. Since the drug was administered in a solution formulation, factors such as drug dissolution and particle size were eliminated, suggesting that liquid viscosity may be a primary influencing factor. Viscosity measurements revealed marked differences among the groups: both the AZT control and AZT + lactose groups showed viscosities similar to water (~1–2 cP), whereas the AZT–pregelatinized starch, AZT + CMS-Na, and AZT + HPMC groups exhibited elevated viscosities of 4.01 cP, 38.4 cP, and 158.2 cP, respectively. In PET imaging studies, the degree of gastric emptying lag followed the order AZT + HPMC > AZT + CMS-Na > AZT + pregelatinized starch > AZT + lactose, which aligns with the rank order of solution viscosity. This correlation supports the hypothesis that increased liquid viscosity contributes to delayed gastric emptying.

Notably, previous reports on viscosity-related gastric emptying have been inconsistent. While some studies using the ^13^C-acetate breath test indicated that high-viscosity meals delay gastric emptying, others observed faster emptying with highly viscous formulations [37,38]. Our experimental data, however, consistently indicate that higher viscosity corresponds to greater retardation of gastric emptying under the present conditions.

To evaluate the impact of gastric emptying on the systemic exposure and pharmacokinetic profile of zidovudine (AZT), rat pharmacokinetic studies were conducted following the administration of AZT alone or in combination with one of the four excipients. The results demonstrated that all excipients reduced the *C_max_* of AZT to varying degrees, with the order of reduction as follows: AZT + CMS-Na > AZT + HPMC ≈ AZT + pregelatinized starch > AZT + lactose. This rank order generally aligned with the viscosity data obtained from the formulations, although the AZT + CMS-Na group exhibited a more pronounced decrease in *C_max_* than anticipated based on viscosity alone. These PK findings are consistent with the PET/CT imaging results, reinforcing the hypothesis that for rapidly metabolized BCS Class I drugs such as AZT, excipients can delay gastric emptying, thereby slowing the rate of absorption and reducing *C_max_*. This correlation is further supported by prior sensitivity analysis of the PBPK model, which identified liquid gastric emptying rate as a critical parameter influencing the absorption of fast-eliminating compounds [5].

Additional permeability studies using Caco-2 monolayers confirmed that none of the four excipients—CMS-Na, HPMC, pregelatinized starch, or lactose—significantly affected the membrane permeability of AZT. The Caco-2 monolayer model, known for its high similarity to human intestinal absorption, has become a standard tool for in vitro permeability assessment. Moreover, zidovudine has been reported to be a highly permeable compound, absorbed primarily via passive diffusion and exhibiting low affinity for P-gp efflux transport [39]. Therefore, the Caco-2 monolayer model was adopted in this study to investigate the effect of zidovudine and zidovudine with excipients on drug permeability. The finding excludes permeability alteration as a contributing factor and strengthens the conclusion that the observed reductions in *C_max_* are primarily mediated by excipient-induced delays in gastric emptying.

Over the past two decades, approximately 180 studies have investigated excipient effects on drug absorption, yet only approximately 10% involved human clinical trials, with the majority employing rodent models or Caco-2 cell systems [40]. While some studies have reported excipient-mediated alterations in absorption, the clinical relevance of these findings must be critically evaluated based on the dosage employed and the predictive validity of the experimental models. Indeed, accumulating evidence suggests that most commonly used excipients do not exert clinically significant effects on the absorption of BCS Class I and III drugs at typically used levels [41,42]. However, the ICH M9 guideline’s vague restriction on excipients that “may affect absorption” for BCS Class I drugs, without establishing a definitive negative list, highlights the imperative for careful, case-by-case assessment of excipient impact during formulation development. Given the substantial species differences, extrapolation of preclinical data to humans warrants careful consideration. Consequently, physiologically based pharmacokinetic (PBPK) or physiologically based biopharmaceutics modeling (PBBM) approaches are recommended for mechanistic prediction and clinical relevance assessment, particularly when dealing with high excipient doses or specific drug-excipient combinations that may pose potential interaction risks [40].

In summary, this study innovatively employed PET/CT imaging to directly quantify the impact of excipients on gastric emptying and further validated PBPK model predictions using complementary animal PK and cellular permeability assays. The direct imaging evidence provides a plausible mechanistic explanation for the occasional bioinequivalence observed with certain rapidly eliminated BCS Class I drugs—namely, that excipients can modulate gastric emptying, thereby transforming it from a physiological-dependent variable into a formulation-dependent variable and eventually altering the rate of drug absorption. These results underscore the importance of considering gastric emptying effects—particularly those related to viscosity-modifying excipients—in the biowaiver evaluation of rapidly eliminated BCS Class I drugs.

Nevertheless, this study has several limitations. First, the effects of excipients were evaluated under fasted conditions using liquid formulations; factors such as food intake, solid dosage forms, and other physiological variables (e.g., dietary composition, caloric content, electrolyte levels, posture, physical activity, and cholecystokinin levels) may also influence gastric emptying and warrant further investigation. Second, whether the excipient-mediated delay in gastric emptying observed in rats translates to humans requires confirmation through clinical trials. The extent of such effects may vary depending on the formulation composition and the specific drug substance. Third, the amount of excipients used in the experiment was relatively larger than the normal range; however, results showed that different types of excipients did have different extents of impact on gastric emptying. Therefore, this study holds significant value. It also pointed out that for rapidly eliminated BCS Class I drugs, in addition to inter-individual physiological differences leading to bioinequivalence, differences between formulation preparations may also lead to bioinequivalence. Finally, CMS-Na, pregelatinized starch, and HPMC are all concentration-dependent, non-Newtonian, shear-thinning polymers whose apparent viscosities decrease with increasing shear rate due to the orientation and disentanglement of macromolecular chains or swollen granules under flow, making single-point measurements at arbitrary rotational speeds inadequate for cross-group comparison. Overall, the primary objective of this study was to qualitatively investigate the impact of different viscosities of pharmaceutical excipients on gastric emptying, and the positive findings obtained herein support the feasibility of our approach. However, future research should be refined to further quantitative studies.

Current BCS-based biowaiver guidelines already recognize that excipients may affect absorption and require justification of excipient sameness/similarity. Our data suggest that for rapidly eliminated BCS Class I drugs, viscosity-modifying excipients may pose a specific *C_max_* bioequivalence risk through the gastric emptying mechanism, meriting attention during formulation development and regulatory review. Nevertheless, our study was conducted in fasted rats using liquid formulations with a single model drug (zidovudine). Whether this would cause BE failure in humans remains to be confirmed in targeted clinical studies. Future studies should expand these investigations to include a broader range of BCS Class I drugs and commonly used excipients, to better understand and mitigate biowaiver risks, and to incorporate excipient viscosity as a modifiable parameter in absorption PBPK models for rapidly eliminated BCS Class I drugs, enabling quantitative risk assessment during formulation development. Systematic evaluation of excipient effects on gastrointestinal physiology will help refine regulatory standards and support more robust biopharmaceutic assessment.

## 5. Conclusions

Based on our team’s prior PBPK modeling, this study innovatively adopted PET/CT imaging to quantitatively assess excipient effects on gastric emptying, complemented by traditional PK and permeability assessments. At the animal level, this study preliminarily validated the hypothesis that excipient selection may cause bioinequivalence even for certain BCS Class I drugs. However, we emphasize that extrapolation from our rat data to human regulatory settings remains hypothesis-generating and requires validation through human PBPK models and clinical BE studies.

Previous studies have shown that for rapidly eliminating BCS Class I drugs, the main reason for bioinequivalence is due to great variations in gastric emptying patterns among individuals and within individuals. That is, it is believed that the gastric emptying process is physiological-dependent rather than formulation-dependent, so generic drugs and original drugs are theoretically the same. However, our research has, for the first time, discovered that gastric emptying can also be formulation-dependent. Our findings demonstrated that certain excipients delay gastric emptying without affecting intestinal permeability, thereby significantly reducing the *C_max_* of rapidly eliminated BCS Class I drugs and posing a risk to biowaiver approval. Specifically, viscosity-enhancing excipients were identified as a key factor slowing gastric emptying and thereby altering *C_max_*. These findings underscore the need for stricter excipient evaluation in biowaiver approaches for rapidly cleared BCS Class I compounds, particularly those containing polymers such as HPMC or CMS-Na. By combining multiple advanced techniques, this work provides multifaceted mechanistic evidence and a new strategic framework for assessing biowaiver risks, supporting more refined regulatory decision-making.

## Figures and Tables

**Table 1:** Summary of animal group and dosing in PET/CT imaging study.

Group	Serial Number	Test Substance and Concentration	Model Probe
1	G1-M-01~ G1-M-06	AZT (3.375 mg/mL)	^18^F-FDG
2	G2-M-01~ G2-M-06	AZT (3.375 mg/mL) + 5% CMS-Na	^18^F-FDG
3	G3-M-01~ G3-M-06	AZT (3.375 mg/mL) + 3% Pregelatinized Starch	^18^F-FDG
4	G4-M-01~ G4-M-06	AZT (3.375 mg/mL) + 4% HPMC	^18^F-FDG
5	G5-M-01~ G5-M-06	AZT (3.375 mg/mL) + 3% Lactose	^18^F-FDG

**Table 2:** %ID values in the stomach of rats after a single intragastric administration of ^18^F-FDG, zidovudine, and four excipients (*n* = 6, mean ± SD).

Time (s)	%ID Values				
	G1	G2	G3	G4	G5
2.5	56.3 ± 34.2	62.0 ± 18.1	80.9 ± 9.61	80.3 ± 11.8	65.7 ± 13.3
10	54.5 ± 34.9	64.9 ± 12.6	80.5 ± 8.34	77.7 ± 19.4	65.9 ± 13.6
22.5	49.9 ± 32.2	65.3 ± 13.8	80.3 ± 6.76	77.3 ± 16.9	62.2 ± 12.1
45	46.8 ± 27.8	71.7 ± 12.1	80.3 ± 8.17	74.1 ± 17.7	61.2 ± 13.4
90	38.0 ± 28.3	67.9 ± 13.6	76.4 ± 10.7	68.1 ± 18.8	58.4 ± 11.3
150	49.9 ± 29.5	62.6 ± 16.3	73.2 ± 16.5	55.6 ± 20.8	54.5 ± 12.3
210	47.5 ± 30.8	56.7 ± 15.0	67.4 ± 18.4	60.8 ± 24.7	51.6 ± 13.9
270	39.2 ± 27.1	52.3 ± 17.4	59.7 ± 26.0	60.5 ± 26.6	48.7 ± 13.9
330	38.8 ± 26.1	51.4 ± 22.0	60.2 ± 21.2	55.7 ± 27.9	47.9 ± 15.1
390	37.3 ± 25.5	44.8 ± 22.5	58.7 ± 23.9	57.4 ± 27.2	44.8 ± 16.3
450	35.1 ± 23.7	45.9 ± 23.5	56.9 ± 20.7	55.9 ± 26.8	42.3 ± 16.9
510	29.6 ± 23.9	42.5 ± 23.6	51.8 ± 24.2	54.1 ± 27.1	39.4 ± 17.8
570	28.5 ± 22.0	40.2 ± 24.7	45.5 ± 21.1	52.2 ± 25.5	36.8 ± 18.0
660	28.2 ± 20.4	37.5 ± 24.5	45.4 ± 21.4	50.3 ± 25.7	35.9 ± 18.2
780	23.2 ± 15.3	31.6 ± 24.8	39.4 ± 15.1	49.6 ± 25.7	31.7 ± 17.1
900	19.7 ± 14.2	33.2 ± 24.9	41.3 ± 13.0	48.5 ± 25.4	29.3 ± 18.2
1020	19.3 ± 13.1	28.9 ± 26.1	37.0 ± 18.3	45.1 ± 23.7	27.1 ± 17.9
1140	17.2 ± 11.3	25.9 ± 23.1	34.6 ± 19.9	43.5 ± 23.8	21.3 ± 14.4
1350	11.3 ± 7.63	23.4 ± 21.5	30.5 ± 17.9	40.8 ± 23.6	17.1 ± 11.0
1650	10.0 ± 7.44	21.4 ± 20.8	27.9 ± 16.7	36.5 ± 20.4	15.6 ± 11.5
2100	7.48 ± 5.41	21.3 ± 23.1	23.4 ± 15.4	31.5 ± 18.7	14.8 ± 11.6
2700	5.90 ± 5.08	19.2 ± 22.0	20.1 ± 19.9	24.4 ± 15.3	12.6 ± 10.3
3300	5.31 ± 4.54	18.5 ± 21.9	20.0 ± 18.6	19.6 ± 12.2	11.0 ± 9.49

**Table 3:** %ID values in the intestine of rats after a single intragastric administration of ^18^F-FDG, zidovudine, and four excipients (*n* = 6, mean ± SD).

Time (s)	%ID Values				
	G1	G2	G3	G4	G5
2.5	30.1 ± 23.9	28.6 ± 20.5	9.62 ± 4.92	10.2 ± 10.2	24.0 ± 5.31
10	29.6 ± 27.0	29.1 ± 20.3	9.11 ± 5.02	12.4 ± 13.9	24.1 ± 5.22
22.5	32.4 ± 30.4	27.1 ± 19.0	11.3 ± 5.02	13.3 ± 15.0	26.8 ± 6.04
45	37.3 ± 24.7	24.1 ± 15.2	12.2 ± 6.87	18.8 ± 16.6	25.1 ± 7.93
90	40.8 ± 25.9	27.4 ± 15.0	15.1 ± 9.87	22.4 ± 20.1	27.5 ± 8.59
150	46.7 ± 28.1	30.4 ± 18.9	18.7 ± 12.7	33.5 ± 24.4	28.1 ± 10.0
210	44.7 ± 28.5	31.3 ± 17.6	23.6 ± 14.8	28.5 ± 27.0	30.1 ± 9.50
270	46.0 ± 29.8	31.6 ± 18.4	27.3 ± 17.5	30.2 ± 28.7	33.3 ± 11.5
330	46.5 ± 27.8	37.3 ± 19.1	28.4 ± 13.1	32.2 ± 27.1	33.9 ± 8.96
390	50.1 ± 22.8	35.8 ± 20.0	31.0 ± 14.7	32.1 ± 27.1	35.5 ± 8.76
450	51.2 ± 20.1	40.7 ± 19.9	28.5 ± 12.2	32.0 ± 26.6	37.3 ± 10.5
510	50.9 ± 19.0	42.1 ± 20.1	31.4 ± 17.4	33.4 ± 26.0	38.5 ± 10.1
570	51.5 ± 16.6	40.9 ± 20.9	30.1 ± 11.1	33.8 ± 25.3	39.9 ± 10.6
660	52.7 ± 15.3	41.1 ± 20.5	31.7 ± 11.0	34.8 ± 23.1	41.9 ± 11.5
780	53.1 ± 12.8	45.8 ± 18.8	32.9 ± 11.9	35.8 ± 21.7	41.2 ± 12.1
900	53.3 ± 6.78	43.4 ± 19.8	29.1 ± 8.23	36.0 ± 19.7	41.3 ± 10.5
1020	50.5 ± 9.03	43.9 ± 19.5	35.2 ± 8.22	37.0 ± 18.5	37.3 ± 9.45
1140	47.4 ± 14.6	42.6 ± 20.0	33.5 ± 4.66	35.4 ± 18.8	43.7 ± 10.6
1350	52.3 ± 9.14	44.5 ± 16.7	32.4 ± 9.40	34.4 ± 16.9	41.8 ± 10.4
1650	51.6 ± 5.95	42.8 ± 17.0	32.6 ± 8.55	32.0 ± 13.2	37.8 ± 9.78
2100	51.1 ± 10.6	39.8 ± 17.7	32.9 ± 10.8	33.5 ± 9.75	33.0 ± 7.00
2700	49.2 ± 16.5	37.9 ± 16.3	32.9 ± 15.1	36.5 ± 11.7	29.1 ± 5.21
3300	49.9 ± 14.9	38.6 ± 17.8	31.0 ± 15.7	35.9 ± 9.31	24.8 ± 5.02

**Table 4:** Fitted gastric emptying rate constants (*k*) of each group after a single gavage of zidovudine and four excipients in rats.

Group	Drug	Rat Liquid Gastric Emptying Rate Constant (h^−1^)
G1	AZT	4.01
G2	AZT + CMS-Na	2.3
G3	AZT + Pregelatinized Starch	2.67
G4	AZT + HPMC	2.09
G5	AZT + Lactose	3.3

**Table 5:** Viscosity of the dosing solution.

Group	Speed	Torque	Viscosity
	RPM *	%	cp
AZT	200	10.8	1.1
AZT + CMS-Na	30	38.4	38.4
AZT + Pregelatinized Starch	200	26.7	4.01
AZT + HPMC	10	58.6	158.2
AZT + Lactose	200	11.3	1.2

* RPM: Revolutions Per Minute.

**Table 6:** The pharmacokinetic parameters of AZT after oral administration in rats.

Parameter	AZT	AZT and CMS-Na	AZT and Pregelatinized Starch	AZT and HPMC	AZT and Lactose	*p* Value
*C_max_* (ng/mL)	13,350.00 ± 4417.21	6965.00 ± 3975.17 *	8760.00 ± 2520.79	8658.33 ± 2434.78	12,305.00 ± 5158.62	0.0404
*T_max_* (h)	0.43 ± 0.18	1.24 ± 1.51	0.54 ± 0.33	0.71 ± 0.25	0.44 ± 0.07	0.2793
*AUC*_0–8_ (h × ng/mL)	16,293.54 ± 3061.88	12,869.09 ± 3684.58	14,512.15 ± 6126.07	14,017.13 ± 1709.07	16,072.19 ± 5535.41	0.6262
*AUC*_0–∞_ (h × ng/mL)	16,371.28 ± 3084.18	13,290.45 ± 3468.96	14,626.07 ± 6201.17	14,265.31 ± 1494.01	16,796.64 ± 4660.64	0.5513
*T*_1/2_ (h)	0.62 ± 0.21	1.67 ± 0.7 *	0.57 ± 0.15	0.57 ± 0.2	1.02 ± 0.59	0.0005
*K* (1/h)	1.21 ± 0.35	0.48 ± 0.19 *	1.26 ± 0.25	1.32 ± 0.36	0.83 ± 0.34	0.0002

*K*: Terminal phase elimination constant. * Statistically significant difference compared with AZT group based on Dunnett’s multiple comparison test.

**Table 7:** The Papp values of AZT and four excipients in the Caco-2 cell model.

Group	Dose (μg/mL)	Excipient Dose (μg/mL)	Papp (A-B) (10^−6^ cm/s)	Papp (B-A) (10^−6^ cm/s)	ER
AZT	50		21.8 ± 3.88	35.94 ± 2.05	1.65
AZT and CMS-Na		20	20.67 ± 3.4	34.75 ± 9.02	1.68
AZT and Pregelatinized Starch			21.89 ± 3.61	30.92 ± 5.9	1.41
AZT and HPMC			19.12 ± 5.22	33.98 ± 8.96	1.78
AZT and Lactose		30	18.14 ± 5.1	28.1 ± 4.68	1.55

## Data Availability

The original contributions presented in this study are included in the article/Supplementary Material. Further inquiries can be directed to the corresponding author(s).

## Supplementary Materials

The following supporting information can be downloaded at https://www.mdpi.com/article/10.3390/pharmaceutics18060634/s1, Table S1: Dunnett’s multiple comparisons test results for %ID values in the stomach of rats after a single intragastric administration of ^18^F-FDG, zidovudine and four excipients.; Figure S1: Individual PET gastric emptying exponential fitting;. Figure S2: (A) Distribution of k values. (B) Mean ± SD of k values; Table S2: Spearman correlation analysis for Viscosity, Rat Liquid Gastric Emptying Rate Constant (k), and Rat C_max_; Table S3: The TEER value in Caco-2 cell monolayers after seeding; Table S4: The Papp value of Lucifer yellow and positive drugs; Table S5: Dunnett’s multiple comparisons test results for apparent permeability among groups.

## Abbreviations

The following abbreviations are used in this manuscript:

BCS	Biopharmaceutics Classification System
BE	Bioequivalence
PBPK	Physiologically based pharmacokinetic modeling
HBSS	Hanks’ balanced salt solution
ESI	Electrospray ionization
PET/CT	Positron emission tomography/computed tomography
CMS-Na	Sodium carboxymethyl starch
HPMC	Hydroxypropyl methylcellulose
LC-MS/MS	Liquid chromatography/tandem mass spectrometry
C_max_	Peak plasma concentration
AZT	Zidovudine
API	Active pharmaceutical ingredient
T_max_	Time to reach C_max_
AUC_0–8h_	Area under the plasma concentration–time curve from time zero to 8 h
T_1/2_	Elimination half-life
AUC_0–∞_	Area under the plasma concentration–time curve from time zero to infinity
SOP	Standard of Practice
%ID/g	The percentage of injected dose per gram of tissue
TEER	Transepithelial electrical resistance
PBBM	Physiologically based biopharmaceutics modeling
P-gp	P-glycoprotein
BCRP	Breast cancer resistance protein
T_½_g_	Gastric half-emptying time
