# Supplementary material for: Elucidating the Critical Role of Excipients in Gastric Emptying and Oral Absorption of a Rapidly Eliminated BCS I Drug: Implications from Zidovudine Bioequivalence
Source: Pharmaceutics. 2026 May 22;18(6):634. doi: 10.3390/pharmaceutics18060634 (PMC13306178; doi:10.3390/pharmaceutics18060634)
Supplement: Supplementary file 1 [file pharmaceutics-18-00634-s001.zip › pharmaceutics-4288000-supplementary.pdf]

pharmaceutics

MDPI

Supplementary Materials: Elucidating the Critical Role of Excipients in Gastric Emptying and Oral Absorption of a Rapidly Eliminated BCS I Drug: Implications from Zidovudine Bioequivalence

Yan Lin, Xian Zhang, Fulin Bi, Guangji Wang and Jin Yang

Table S1. Dunnett's multiple comparisons test results for %ID values in the stomach of rats after a single intragastric administration of ^{18}F-FDG, zidovudine and four excipients.

	Mean Diff.	95% CI of diff.	Significant?	P value
G1 vs. G2	−12.61	-20.29 to -4.931	Yes	0.0003
G1 vs. G3	−20.90	-28.67 to -13.14	Yes	<0.0001
G1 vs. G4	−22.64	-30.37 to -14.91	Yes	<0.0001
G1 vs. G5	−8.556	-15.74 to -1.376	Yes	0.0135

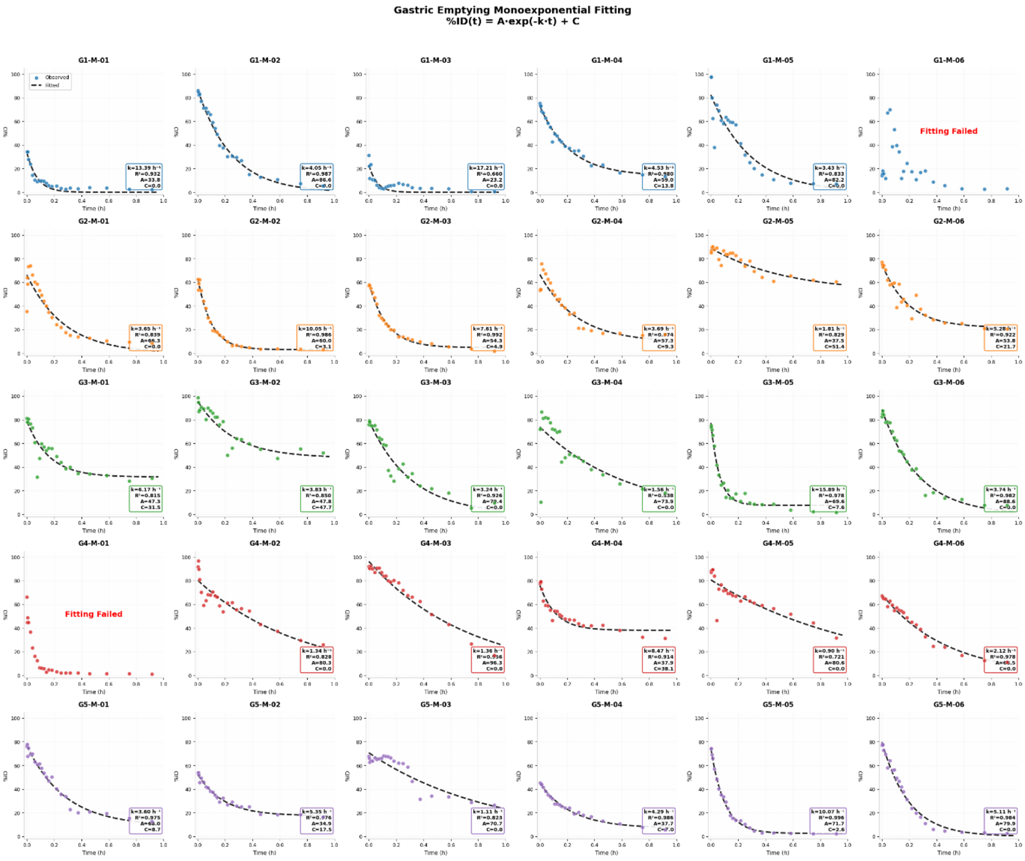

Figure S1. Individual PET gastric emptying exponential fitting.

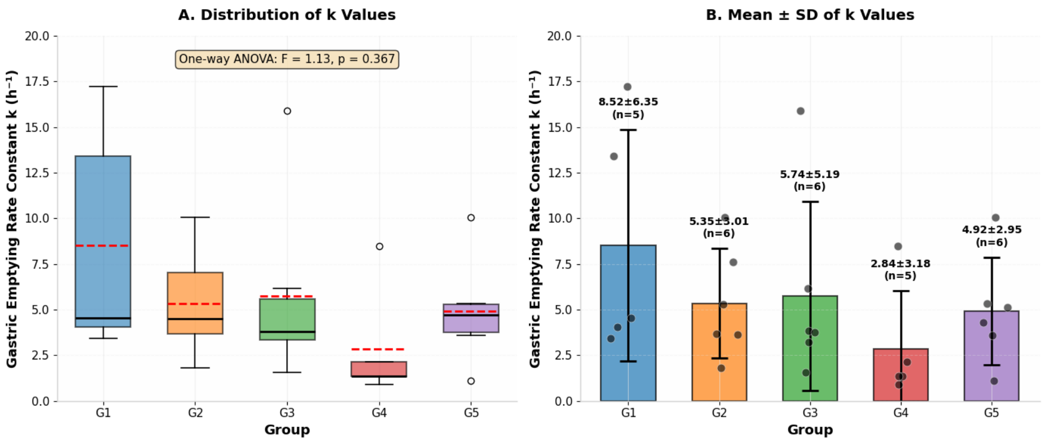

Figure S2. (A) Distribution of *k* values. (B) Mean±SD of *k* values.

Table S2. Spearman correlation analysis for Viscosity, Rat Liquid Gastric Emptying Rate Constant (*k*), and Rat C_{max}.

	**Viscosity vs. *k***	**Viscosity vs. C_{max}**
p value	0.0167	0.0833
Significant? (alpha = 0.05)	Yes	No

Table S3. The TEER value in Caco-2 cell monolayers after seeding (Mean ± SD, *n* = 42)

**Time of cultivation（day）**	**TEER (Ω·cm²)**
2	67.7 ± 10.2
8	204.5 ± 13.4
14	712.5 ± 51.1
17	587.3 ± 31.1
21	520.1 ± 29.2

Table S4. The P_{app} value of Lucifer yellow and positive drugs (Mean±SD，*n* = 3).

**Group**	**Dose**	**$P_{app\ (A\text{-}B)}$（cm/s）**
Propranolol		$25.89 \times 10^{-6} \pm 7.78 \times 10^{-6}$
Metoprolol	50 μMol	$14.76 \times 10^{-6} \pm 2.69 \times 10^{-6}$
Atenolol		$2.65 \times 10^{-6} \pm 0.67 \times 10^{-6}$
Lucifer yellow	300 μMol	$2.73 \times 10^{-8} \pm 0.87 \times 10^{-8}$

Table S5. Dunnett's multiple comparisons test results for apparent permeability among groups.

	**AZT + CMS-Na vs. AZT**	**AZT + HPMC vs. AZT**	**AZT + Pregelatinized Starch vs. AZT**	**AZT + Lactose vs. AZT**
Papp (A→B)	0.9912	0.8529	>0.9999	0.6880
Papp (B→A)	0.9980	0.9880	0.7680	0.4484

https://doi.org/10.3390/xxxxx
